# Structural and kinetic analysis of an MsrA–MsrB fusion protein from *Streptococcus pneumoniae*

**DOI:** 10.1111/j.1365-2958.2009.06680.x

**Published:** 2009-05

**Authors:** Young Kwan Kim, Youn Jae Shin, Won-Ho Lee, Hwa-Young Kim, Kwang Yeon Hwang

**Affiliations:** 1 Division of Biotechnology, College of Life Sciences and Biotechnology, Korea UniversitySeoul 136-701, Korea; 2 Department of Biochemistry and Molecular Biology, Aging-associated Vascular Disease Research Center, Yeungnam University College of MedicineDaegu 705-717, Korea

## Abstract

Methionine sulphoxide reductases (Msr) catalyse the reduction of oxidized methionine to methionine. These enzymes are divided into two classes, MsrA and MsrB, according to substrate specificity. Although most MsrA and MsrB exist as separate enzymes, in some bacteria these two enzymes are fused to form a single polypeptide (MsrAB). Here, we report the first crystal structure of MsrAB from *Streptococcus pneumoniae* (*Sp*MsrAB) at 2.4 Å resolution. *Sp*MsrAB consists of an N-terminal MsrA domain, a C-terminal MsrB domain and a linker. The linker is composed of 13 residues and contains one 3_10_-helix and several hydrogen bonds interacting with both MsrA and MsrB domains. Interestingly, our structure includes the MsrB domain complexed with an SHMAEI hexa-peptide that is the N-terminal region of neighbouring MsrA domain. A kinetic analysis showed that the apparent *K*_m_ of *Sp*MsrAB for the *R*-form-substrate was 20-fold lower than that for the *S*-form substrate, indicating that the MsrB domain had a much higher affinity for the substrate than the MsrA domain. Our study reveals the first structure of the MsrAB by providing insights into the formation of a disulphide bridge in the MsrB, the structure of the linker region, and the distinct structural nature of active site of each MsrA and MsrB domain.

## Introduction

Methionine (Met) is one of the most oxidation-sensitive amino acids. Oxidation of Met to methionine sulphoxide (Met-SO) damages the proteins and can alter protein function (Hoshi and Heinemann, 2001; Weissbach *et al*., 2002; Moskovitz, 2005; Kim and Gladyshev, 2007). However, these damaged proteins can be reversed by the repair enzymes, methionine sulphoxide reductases (Msr). Msr enzymes are essential to protect cells, such as bacteria, mammals and plants, against oxidative stress (Skaar *et al*., 2002; Alamuri and Maier, 2004; Bechtold *et al*., 2004; Kantorow *et al*., 2004), and are also implicated in ageing and neurodegenerative diseases (Gabbita *et al*., 1999; Moskovitz *et al*., 2001; Ruan *et al*., 2002; Friguet, 2006; Wassef *et al*., 2007). Two distinct Msr enzymes have been classified for the reduction of Met-SO: MsrA reduces the *S*-form of Met-SO and MsrB reduces the *R*-form (Brot *et al*., 1981; Sharov and Schoneich, 2000; Grimaud *et al*., 2001). MsrA and MsrB have their active sites that display an essentially mirror-image-like relationship, reflecting the observed stereo-specificity of these enzymes (Lowther *et al*., 2002). The MsrA and MsrB sequences show no homology to each other. However, enzymatic studies demonstrate that both the enzymes share a common catalytic mechanism based on sulphenic acid chemistry involving two (or three) cysteine residues (Boschi-Muller *et al*., 2000; Kumar *et al*., 2002; Antoine *et al*., 2003; Olry *et al*., 2004; Kauffmann *et al*., 2005).

Most organisms contain MsrA and MsrB typically as separate enzymes. However, MsrA and MsrB exist as domains in a single fused protein (MsrAB) in some bacteria such as *Streptococcus pneumoniae*, *Neisseria gonorrhoeae* and *Haemophilus influenza* (Kryukov *et al*., 2002; Delaye *et al*., 2007). Why some bacteria possess the fusion protein instead of separate MsrA and MsrB forms is not clear. Analysis of the sequence alignment of MsrA, MsrB and MsrAB reveals that the active sites are conserved in each protein. To date, individual MsrA and MsrB structures from eight species have been reported (Lowther *et al*., 2000; 2002; Tête-Favier *et al*., 2000; Taylor *et al*., 2003; Rouhier *et al*., 2007; Ranaivoson *et al*., 2008) or deposited (1XM0, 3CXK and 3CEZ). However, there is no structural information on MsrAB. Compared with either the MsrA or MsrB structure, MsrAB structure may be somewhat different since a linker region exists between the MsrA and MsrB domains.

In this study, we report the first crystal structure of MsrAB from *S. pneumoniae* (*Sp*MsrAB). The *Sp*MsrAB consists of an N-terminal MsrA domain (*Sp*MsrA), a C-terminal MsrB domain (*Sp*MsrB) and a linker. The linker, *iloop*, contains conserved residues that participate in significant hydrogen bonds perhaps to maintain structural stability. Interestingly, our structure includes an *Sp*MsrB complex with a hexa-peptide (SHMAEI) that is the N-terminal region of neighbouring *Sp*MsrA through crystal packing. This is the first report of a complex form with a peptide including the product Met in the active site of MsrB structure. Kinetic analysis revealed that the apparent *K*_m_ value of *Sp*MsrAB for Met-*R*-SO is 20-fold lower than that for Met-*S*-SO and the catalytic efficiency (*k*_cat_/*K*_m_) for the *R*-form is sevenfold higher than that for the *S*-form.

## Results and discussion

### Overall structure and comparison of *Sp*MsrAB

**Fig. 1:**
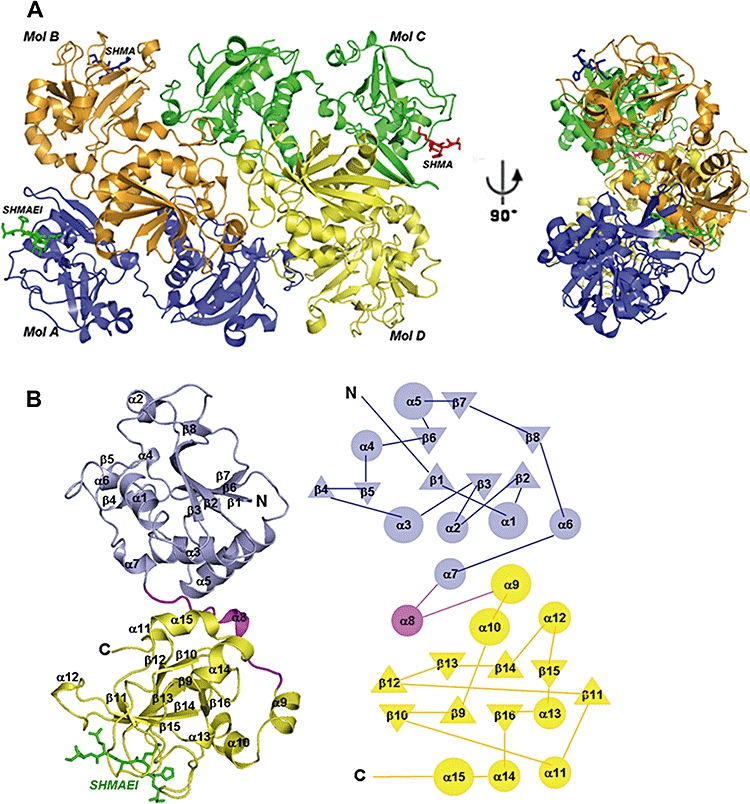
Overall structures of *Sp*MsrAB. A. In the crystallographic orthorhombic form, four molecules of *Sp*MsrAB show two conformations in an asymmetric unit. Mol A and Mol C are the same conformation while Mol B and Mol D are the other conformation. SHMAEI and SHMA peptides are shown by stick models. The SHMAEI hexa-peptide in Mol A is originated from the N-terminal region of Mol D in the neighbouring unit cell. The molecules that donate the SHMA in Mol B and Mol C are not clear. B. Ribbon diagram of the structure of the *Sp*MsrAB–SHMAEI hexa-peptide complex, with individual elements of secondary structure labelled. All figures were created using the pymol (http://pymol.sourceforge.net/) (left). Topology diagram of *Sp*MsrAB was drawn with tops (Westhead *et al*., 1999). α-Helices (circles) are labelled α1–α15 and β-strand (triangles) are labelled β1–β16 (right).

The orthorhombic structure of *Sp*MsrAB was determined using the method of single-wavelength anomalous dispersion (SAD) to a resolution of 2.4 Å with seleno-Met. Phasing and refinement statistics are shown in Table 1. The asymmetric unit contains four molecules that show two conformations (Fig. 1A and Fig. S1). Molecule A (Mol A) and molecule C (Mol C) are the same conformation (conformation 1) while molecule B (Mol B) and molecule D (Mol D) are the other conformation (conformation 2). Interestingly, Mol A contains an SHMAEI hexa-peptide and Mol B and Mol C have an SHMA peptide in their MsrB domains, but there appears no peptide bound in Mol D. This structure results from the crystal packing. Thus far, no MsrB structures in complex with a peptide substrate (or product) have been reported. The SHMAEI hexa-peptide in Mol A was donated from the symmetric Mol D molecule in the neighbouring unit cell (Figs S1 and S2). The Ser and His are originated from the His-tag following thrombin treatment cleavage in the protein purification. The Met, Ala, Glu and Ile of the hexa-peptide are identical to the N-terminal residues of the native *Sp*MsrAB sequence. The molecules donating SHMA peptides bound in Mol B and Mol C are not clear.

**Table 1:** Data collection and refinement statistics.

	*Sp*MsrAB	
	SeMet-peak	Native
Data collection		
Space group	P2_1_2_1_2	P2_1_2_1_2
Cell dimension, *a*, *b*, *c* (Å)	158.8 165.0 77.9	158.5 165.5 77.3
Molecules per AU	4	4
Wavelength (Å)	0.97950	1.00000
Resolution range (Å)	20.0–2.70 (2.80–2.70)	20.0–2.4 (2.48–2.40)
No. of measured reflections	1 533 200	1 036 517
No. of unique reflections	55 599	90 336
Completeness (%)	99.6 (98.8)	91.9 (77.5)
Average *I*/σ(*I*)	26.9 (3.5)	9.3 (1.3)
*R*_merge_^a^ (%)	11.1 (36.8)	10.5 (46.5)
Refinement		
Resolution range (Å)	20.0–2.7	20.0–2.4
No. of reflections (work/test)	56 761 (51 027/5734)	77 162 (70 101/7061)
*R*_work_^b^/*R*_free_^c^	22.2/28.6	23.9/28.2
B-factors (Å^2^) (protein/solvent)	44.0/38.0	47.3/50.4
No. of atoms (protein/ligand/water)	10 088/92/413	10 104/107/305
Root mean square deviations		
Bond lengths (Å)	0.008	0.007
Bond angles (degree)	1.3	1.4
Ramachandran plot		
Most favoured (%)	82.9	87.3
Additionally allowed (%)	16.6	12.6
Generously allowed (%)	0.5	0.1
Disallowed (%)	0.1	0.0

a *R*_merge_ = Σ_*h*_Σ_*j*_|〈*I*〉_*h*_ − *I_hj_*|/Σ_*h*_Σ_*j*_|*I_hj_*, where 〈*I*〉_*h*_ is the mean intensity of symmetry-equivalent reflections.

b *R*_work_ = Σ_*h*_|*F*_o_ − *F*_c_|/Σ_*h*_|*F*_o_|, where *F*_o_ and *F*_c_ are the observed and calculated structure factor amplitudes of reflection *h*.

c *R*_free_ is the same as *R*_work_, but calculated on the reflections set aside from refinement.

**Fig. 2:**
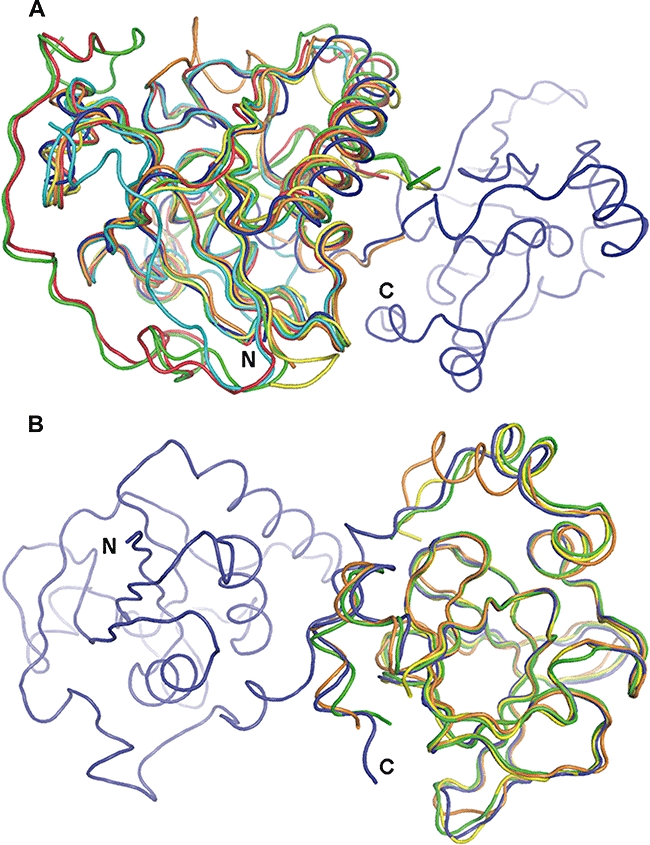
Structural comparison of MsrAs and MsrBs. A. Comparison of MsrAs. The backbone models for *E. coli*, *Bos taurus*, *Mycobacterium tuberculosis*, *Neisseria meningitides* (3BQH), *Popular trichocarpa* and *Streptococcus pneumoniae* are shown in green, red, yellow, orange, cyan and blue respectively. B. Comparison of MsrBs. The backbone models for *Bacillus subtilis*, *Neisseria gonorrhoaea*, *Burkholderia pseudomallei* (3CXK) and *S. pneumoniae* are shown in orange, green, yellow and blue respectively.

**Fig. 3:**
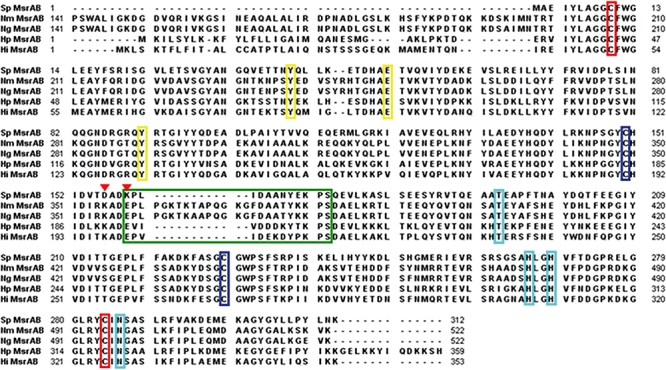
Multiple sequence alignment of MsrABs. Multiple sequence alignment of *Hp*MsrAB, *Hi*MsrAB, *Nm*MsrAB, *Ng*MsrAB and *Sp*MsrAB is shown (Hi, *Haemophilus influenza*; Hp, *Helicobacter pylori*; Nm, *Neisseria meningitides*; Ng, *Neisseria gonorrhoae*)*.* Sequence alignment was accomplished with BioEdit (http://www.mbio.ncsu.edu/BioEdit/bioedit.html). In the sequence alignment, red and blue box indicate the catalytic and recycling cysteine residues respectively; yellow and cyan box indicate the conserved residues participating in the catalytic mechanisms of MsrA and MsrB respectively. The linker region is shown in green box and Asp-156 and Lys-159 are shown as an inverted red triangle.

The monomeric structure of *Sp*MsrAB has the overall dimensions of approximately 74.5 × 37.5 × 39.7 Å^3^ and consists of two major domains, MsrA (residues 1–158) and MsrB (residues 172–312), and a linker domain (residues 159–171) (Fig. 1B). The N-terminal *Sp*MsrA and C-terminal *Sp*MsrB domains are structurally similar to the previously reported individual MsrA and MsrB proteins respectively. The N-terminal MsrA domain of *Sp*MsrAB is composed of eight rolled mixed β-strands (β1–β8) and seven α-helices (α1–α7), similar to *Bos taurus* MsrA (*Bt*MsrA) (Lowther *et al*., 2000), *Escherichia coli* MsrA (*Ec*MsrA) (Tête-Favier *et al*., 2000), *Mycobacterium tuberculosis* MsrA (*Mt*MsrA) (Taylor *et al*., 2003), *Neisseria meningitides* MsrA (*Nm*MsrA) (Ranaivoson *et al*., 2008) and *Populus trichocarpa* MsrA (*Pt*MsrA) (Rouhier *et al*., 2007) (Fig. 2A). The MsrB domain of *Sp*MsrAB is composed of seven antiparallel β-strands (β9–β16) and seven α-helices (α9–α15), similar to the structures of MsrB from *N. gonorrhoeae* (*Ng*MsrB) (Lowther *et al*., 2002), *Burkholderia pseudomallei* (*Bp*MsrB; 3CEZ, 3CXK) and *Bacillus subtilis* (*Bs*MsrB; 1XM0). We also determined the crystal structure of *Bs*MsrB (3E0O) for comparison of *Sp*MsrB (Fig. S3 and Table S1; Park *et al*., 2008). It should be noted that the crystal structures of *Bp*MsrB (3CEZ, 3CXK) and the NMR structure of *Bs*MsrB (1XM0) had not been reported when we initiated this study. Our crystal structure of *Bs*MsrB was found to be identical to the NMR *Bs*MsrB structure and was further used for structure comparison. Comparison of *Sp*MsrB with *Bp*MsrB, *Bs*MsrB and *Ng*MsrB revealed that *Sp*MsrB is more highly conserved than *Sp*MsrA (Fig. 2B). A sequence alignment analysis indicated that *Sp*MsrAB shows a high sequence identity to other bacterial MsrABs: 52% (to *N. meningitides*), 53% (to *N. gonorrhoeae*), 60% (to *Helicobacter pylori*) and 59% (to *H. influenza*) (Fig. 3). These bacterial MsrABs are linked by a hinge region; *Ng*MsrAB and *Nm*MsrAB contain additional 12 residues in their linker regions compared with those of other species. However, the role of the linker in MsrABs is not known. The linker, *iloop*, of *Sp*MsrAB consists of 13 residues (residues 159–171) and, interestingly, contains one 3_10_-helix and several hydrogen bonds to both the MsrA and MsrB domains that could restrict the positions of both domains.

### Structural positions of critical cysteines in catalysis in *Sp*MsrAB

**Fig. 4:**
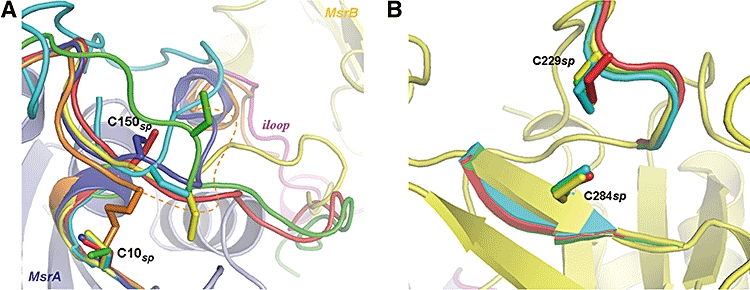
Structural positions of active-site cysteine residues. A. The positions of cysteines in *Sp*MsrA. The catalytic Cys-10 and recycling Cys-150 are labelled. The stick models for the active-site amino acids for *Ec*MsrA (green), *Bt*MsrA (red), *Mt*MsrA (yellow), *Nm*MsrA (orange), *Pt*MsrA (cyan) and *Sp*MsrA (blue) are superimposed. For *Nm*MsrA (3BQH), the approximate position of unresolved sequence was added and connected with the dotted line. B. The positions of cysteines in *Sp*MsrB. The catalytic Cys-284 and recycling Cys-229 are labelled. The stick models for the active-site amino acids for *Bs*MsrB (red), *Ng*MsrB (cyan), *Bp*MsrB (green; 3CXK) and *Sp*MsrB (yellow) are superimposed.

Cys-10 and Cys-284 are charged for catalytic attack of Met-*S*-SO and Met-*R*-SO respectively. Cys-150 and Cys-229 (called recycling cysteines) interact with the oxidized catalytic cysteines (sulphenic acid intermediates), respectively, to form disulphide bonds. Cys-10 and Cys-150 of *Sp*MsrA were compared with those of *Ec*MsrA, *Bt*MsrA, *Mt*MsrA, *Pt*MsrA and *Nm*MsrA (Fig. 4A). The structural positions of Cys-10 and Cys-150 were similar to those of other MsrAs. As expected from the structures of *Ec*MsrA, *Bt*MsrA, *Mt*MsrA and *Pt*MsrA, the catalytic Cys-10 of *Sp*MsrA is remote from the recycling Cys-150 which is located within the flexible loop (residues 145–153). The distances between the two cysteine residues are 8.0 Å (*Sp*MsrA), 8.6 Å (*Bt*MsrA), 12.8 Å (*Ec*MsrA), 6.8 Å (*Mt*MsrA) and 7.1 Å (*Pt*MsrA). To form a disulphide bridge, the two catalytic and recycling cysteine residues in such a long distance may undergo conformational changes that bring the residues within approximately 3.0 Å of each other, as shown in the structure of *Nm*MsrA (Ranaivoson *et al*., 2008). As shown in Fig. 4B, unlike *Sp*MsrA, the catalytic Cys-284 and recycling Cys-229 of *Sp*MsrB are structurally identical to those of the *Ng*MsrB, *Bp*MsrB and *Bs*MsrB. The distance (4.1 Å) between Cys-284 and Cys-229 is sufficient to enable formation of a disulphide bridge. To form a disulphide bridge, the β-carbon (Cβ) of Cys-284 must rotate towards the substrate Met-*R*-SO and the angle of the Cα of Cys-229 must change. These observations suggest that MsrB can easily form a disulphide bridge once the protein binds a substrate.

### Complex formation with the SHMAEI hexa-peptide of *Sp*MsrAB

**Fig. 5:**
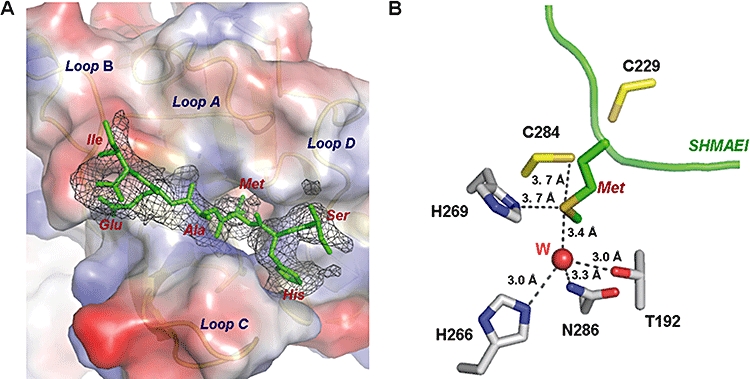
The *Sp*MsrB–SHMAEI complex. A. A complex structure of the *Sp*MsrB–SHMAEI hexa-peptide. Four loops that surround the SHMAEI hexa-peptide are shown in surface model and the SHMAEI hexa-peptide is shown by 2*F*_o_ − *F*_c_ electron density map at 1.0 σ. B. Interaction in the *Sp*MsrB–SHMAEI hexa-peptide complex. The detail interaction between the Met of SHMAEI hexa-peptide and the active-site residues of *Sp*MsrB is displayed. The water molecule is shown as a red sphere. The residues involving the interactions are shown in stick models. The Met residue is shown as a green stick model.

Most MsrA structures contain a complex with either a small molecule such as dithiothreitol, mercaptoethanol and arsenate group (Lowther *et al*., 2000; Tête-Favier *et al*., 2000; Rouhier *et al*., 2007), or a Met residue through a crystal packing (Taylor *et al*., 2003), or even a Met-SO substrate (3BQF) (Ranaivoson *et al*., 2008) in their active pocket. *Ng*MsrB structure also includes a cacodylate molecule in its active site (Lowther *et al*., 2002). Interestingly, as mentioned, our *Sp*MsrAB structure reveals a complex between the active pocket of *Sp*MsrB and the SHMAEI hexa-peptide, and is the first report of the structure of an MsrB complex with the product Met. The hexa-peptide is surrounded by four loops, A, B, C and D (Fig. 5A). The structure of the *Sp*MsrB complex indicates that the catalytic Cys-284 faces the sulphur atom of the Met residue. His-269 and one water molecule also interact with the sulphur atom of the Met residue. Thr-192, His-266 and Asn-286 are stabilized by their interaction with water (Fig. 5B). The Thr-192, His-266, His-269 and Asn-286 have been proposed to be important for MsrB catalysis and these residues are well conserved in all MsrBs. The structurally ordered water molecule, which interacts with Thr-192, Asn-286 and Met-SO during the enzymatic reaction, is also suggested to play a key role in the enzymatic reaction of MsrB as shown in the structure of *Ng*MsrB (Lowther *et al*., 2002). Our complex structure with the SHMAEI peptide supports the catalytic mechanism model of MsrB proposed earlier (Lowther *et al*., 2002).

**Fig. 6:**
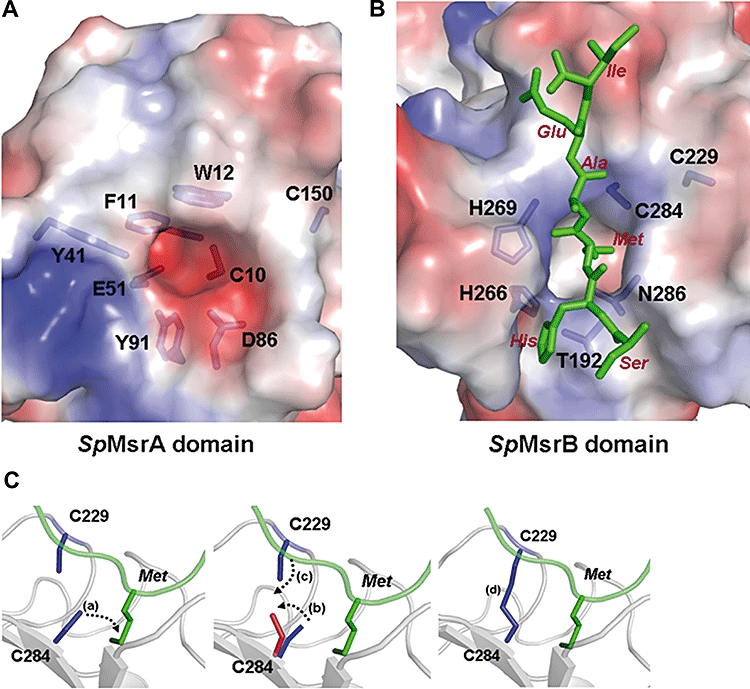
Surface models of active pockets of MsrA and MsrB domains and MsrB catalytic mechanism model. A. A surface model of *Sp*MsrA active pocket. The stick models of residues (except Cys-10 and Cys-150) show the steric hindrance produced when a substrate attempts to access the pocket of *Sp*MsrA. The active pocket is negatively charged. B. A surface model showing the *Sp*MsrB complex. The green stick model is the SHMAEI hexa-peptide and the blue stick models are the residues that interact with the hexa-peptide. The active pocket is positively charged. C. A model for MsrB catalytic mechanism. When MsrB accesses a substrate, catalytic Cys-284 attacks the sulphur atom of its substrate (i.e. Met-*R*-SO) (a). Recycling Cys-229 and the oxidized Cys-284 (sulphenic acid form) change their positions (b) to establish a disulphide bridge (c). A complete disulphide bridge is formed in *Sp*MsrB (d). The model shows *Sp*MsrB and the SHMAEI hexa-peptide. Cys-229 and Cys-284 are shown in stick models (blue). The red stick model represents the shift of Cys-284 after MsrB attacks a substrate and the green stick model is the SHMAEI hexa-peptide.

Similar to the general structural features of the MsrBs, the structure of *Sp*MsrB demonstrates that the active site is exposed to solvent and that its substrate, Met-*R*-SO, can readily form a complex with the enzyme. The active-site pocket of *Sp*MsrA is negatively charged while that of *Sp*MsrB is positively charged, similar to the previously known MsrA and MsrB structures. These opposite charge distributions in the substrate binding pockets of *Sp*MsrA and *Sp*MsrB may contribute to a factor for discriminating their stereo-specific substrates, Met-*S*-SO and Met-*R*-SO, in which the positions of oxygen of the sulphoxide moiety are different from each other. Unlike *Sp*MsrB, the residues forming the active-site pocket in *Sp*MsrA are so bulky that the steric hindrance may be produced from the aromatic ring structures of Phe-11, Trp-12 and Tyr-91 when a substrate attempts to access the active pocket (Fig. 6A). Despite no significant conformational changes between the reduced *Nm*MsrA (3BQE) and the complexed form with a Met-SO substrate (3BQF) (Ranaivoson *et al*., 2008), the flanked loop, including β4 and β5, of the active pocket appears to be somewhat different between these two forms (Fig. S4). It is more ‘open’ state in the reduced form than in the complexed form. This flanked loop of the *Sp*MsrA was more ‘closed’ state than those of the complexed *Nm*MsrA and *Mt*MsrA forms (Fig. S4). Taken together, the results suggest the ‘closed’ nature of the MsrA active site relative to the ‘open’ MsrB active site in *Sp*MsrAB. However, there is a possibility that the crystal contacts and packing of the four molecules within the crystal lattice may have preferentially stabilized the ‘closed’ state of active site of *Sp*MsrA domain.

As described above, the formation of the product Met–*Sp*MsrB complex is correlated to the kinetics of *Sp*MsrAB. The surface model of *Sp*MsrB demonstrates that the SHMAEI hexa-peptide fits within the active-site pocket (Fig. 6B). Based on these results, we propose a structural model for the disulphide bridge formation in MsrB (Fig. 6C). When *Sp*MsrB accesses Met-*R*-SO, (i) the catalytic Cys-284 attacks the sulphur atom of Met-*R*-SO and then a sulphenic acid intermediate is formed, (ii) once the product Met is released, the Cβ angle of Cys-284 rotates towards the recycling Cys-229, (iii) the Cys-229 also moves towards the Cys-284 and (iv) a disulphide bridge forms between these residues. Finally, the oxidized MsrB is associated with thioredoxin to reduce the disulphide bond.

### Specific activity and kinetic analysis

The specific activities and kinetic parameters of *Sp*MsrAB and *Bs*MsrB are summarized in Table 2. HPLC for kinetic analysis and the preparation of dabsyl-Met-*R*-SO and dabsyl-Met-*S*-SO were conducted according to established procedures (Minetti *et al*., 1994; Kumar *et al*., 2002; Etienne *et al*., 2003). The MsrB activity of *Sp*MsrAB was slightly higher than its MsrA activity at 200 μM substrate. However, the apparent *k*_cat_ value for dabsyl-Met-*R*-SO was threefold lower than that for dabsyl-Met-*S*-SO. The total Msr activity was consistent with the sum of the MsrA and MsrB activities. The specific MsrA and MsrB activities of *Sp*MsrAB were similar to those of mammalian MsrA and MsrB (Kim and Gladyshev, 2004; Kim and Gladyshev, 2005). The specific activity of *Bs*MsrB was slightly lower than the MsrB activity of *Sp*MsrAB and of mammalian MsrBs. *K*_m_ values of *Sp*MsrAB were 0.86 mM for dabsyl-Met-*S*-SO and 0.038 mM for dabsyl-Met-*R*-SO. These data suggest that *Sp*MsrAB can efficiently catalyse the Met-*R*-SO to Met reaction at low substrate concentrations. *Bs*MsrB also has a low *K*_m_ value, similar to that of *Sp*MsrAB for dabsyl-Met-*R*-SO. The catalytic efficiency (*k*_cat_/*K*_m_) of *Sp*MsrAB for dabsyl-Met-*R*-SO is sevenfold higher than that for dabsyl-Met-*S*-SO. These results suggest that the MsrB domain contains a more efficient catalytic activity than the MsrA domain in the bacterial MsrAB. Moreover, the higher affinity for the *R*-form of the substrate may have been correlated with the *Sp*MsrB complex formation with the SHMAEI hexa-peptide in the crystal structure.

**Table 2:** Specific activities and kinetic parameters of *Sp*MsrAB.

Protein	Substrate	Specific activity [nmol min^−1^ (mg protein)^−1^]	*K*_m_ (mM)	*k*_cat_ (s^−1^)	*k*_cat_/*K*_m_ (M^−1^ s^−1^)	Reference
*S. pneumoniae* MsrAB	Met-*S*-SO	250 ± 7	0.86 ± 0.05	0.80 ± 0.04	930 ± 10	This study
	Met-*R*-SO	327 ± 3	0.038 ± 0.002	0.24 ± 0.01	6320 ± 150	
	Met-*R*,*S*-SO	580 ± 7	NA	NA	NA	
*B. subtilis* MsrB	Met-*R*-SO	264 ± 14	0.041 ± 0.002	0.10 ± 0.005	2440 ± 70	This study
Mouse MsrA	Met-*S*-SO	238 ± 26	0.34 ± 0.04	0.28 ± 0.02	820 ± 50	Kim and Gladyshev (2005)
Mouse MsrB2	Met-*R*-SO	353	0.17	0.23	1350	Kim and Gladyshev (2004)
Human MsrB3	Met-*R*-SO	423	2.9	2.29	790	Kim and Gladyshev (2004)

The specific activities and kinetic parameters were determined using dabsylated substrates as described in *Experimental procedures*.

NA, not assayed.

### The linker region of *Sp*MsrAB

**Fig. 7:**
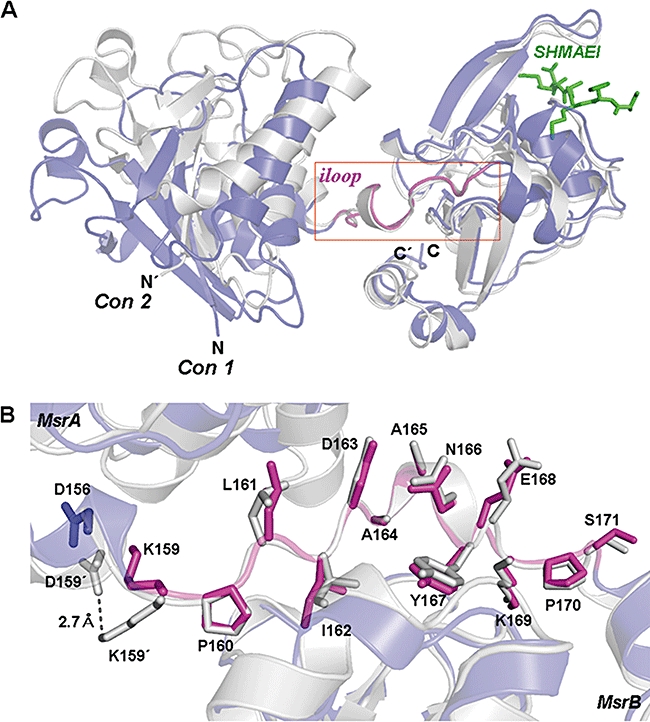
The movement of MsrA and the hinge loop, *iloop*, motion of *Sp*MsrAB. A. Two conformations appeared in the crystallographic asymmetric unit. The position of *Sp*MsrA moves approximately 10 Å. *Con 1* and *Con 2* show the distinct positions of the *Sp*MsrA domain in an asymmetric unit when superposed by optimizing the linker regions of two conformations. The blue is shown as a conformation 1 (*Con 1*) and the white as a conformation 2 (*Con 2*). The N- and C-terminal of *Con 2* is presented with N′ and C′ respectively. The red box is a linker region. The hexa-peptide SHMAEI from *Con 1* is shown in green stick model. B. The interaction change of Lys-159 between two conformations (*Con 1* and *Con 2*). Asp-156 and Lys-159 of *Con 2* is presented with Asp-156′ and Lys-159′ respectively. The magenta is the linker region of *Con 1* while the white is the linker of *Con 2*. The amino acids of linker region are shown as the stick models.

*Sp*MsrA and *Sp*MsrB form a single protein joined by a linker. Why some bacteria contain an MsrAB rather than separate MsrA and MsrB is not clear, and raises a question as to whether these two domains behave differently in the fused protein. In this work, we first demonstrated the structure of MsrA–MsrB fusion protein including the linker region (see also the electron density map of the linker region in Fig. S5). Unexpectedly, two structural conformations of *Sp*MsrAB were revealed in an asymmetric unit. We compared these two conformations and found that *Sp*MsrA occurs in two dramatically distinct conformations relative to the centre of the *iloop*, while *Sp*MsrB forms a complex with Met (Fig. 7A). *Sp*MsrB is complexed with the SHMAEI hexa-peptide, and the movement of *Sp*MsrB is therefore restricted. In contrast, *Sp*MsrA may move over approximately 10 Å. As described above, the hinge loop *iloop*, which connects MsrA and MsrB, is composed of residues 159–171. The movement of Lys-159 is remarkable in the two conformations of the structure (Fig. 7B).

As mentioned, the *iloop* contains one 3_10_-helix and several hydrogen bonds interacting with residues at both the MsrA and MsrB domains. The interacting hydrogen bonds are summarized in Table 3. We also found changes of hydrogen bonds between Msr domains and *iloop* in the two conformations (Table 3). An outstanding change in hydrogen bonds is a new bond formation between Asp-156 of *Sp*MsrA and Lys-159 of *iloop* in the conformation 2 (Fig. 7B). This interaction between Asp-156 and Lys-159 may induce (or result from) the distinct movement of *Sp*MsrA. These results suggest that the movement of MsrA domain in other MsrABs may also occur while MsrB domain movement may be restricted relative to the central *iloop*, although the Asp-156 and Lys-159 of *Sp*MsrAB are not conserved in other MsrABs. According to the sequence alignment, other MsrABs have Lys and Glu residues in these positions (Fig. 3), but these two residues are likely to interact with each other as well. To verify the role of these residues, further studies will be needed.

**Table 3:** The hydrogen bonds and their changes between Msr domains and *iloop* in the two conformations.

Hydrogen bond	Conformation 1	Conformation 2
*MsrA–iloop*		
Arg-65 (NH2)–Asp-163 (Oδ1)	2.9 Å	3.0 Å
Arg-65 (Nε)–Asp-163 (Oδ2)	2.5 Å	2.8 Å
Arg-73 (NH1)–Lys-159 (O)	2.8 Å	3.4 Å
Arg-73 (NH2)–Pro-160 (O)	3.2 Å	2.9 Å
Asp-156 (Oδ1)–Lys-159 (Nζ)	–	2.7 Å
*iloop–MsrB*		
Ser-171 (Oγ)–Val-174 (Cγ1)	2.9 Å	3.0 Å
Ser-171 (O)–Leu-175 (N)	3.2 Å	3.1 Å
Lys-169 (Nζ)–Gln-188 (Oε1)	2.8 Å	–
Tyr-167 (O)–Ser-262 (Oγ)	2.6 Å	2.6 Å
Ile-162 (O)–Arg-261 (Nη2)	2.9 Å	2.9 Å
Tyr-167 (OH)–Tyr-305 (OH)	2.9 Å	2.9 Å

–, none.

The linker of *Sp*MsrAB may stabilize the positions of the MsrA and MsrB domains relative to each other, despite the fact that two distinct conformations are observed in an asymmetric unit. In regard to the occurrence of two dramatically different MsrA conformations, the *Sp*MsrA may have another function in the cells in addition to the catalytic function.

In summary, we determined the first crystal structure of *Sp*MsrAB at 2.4 Å resolution. First, we suggest that the *iloop* region of *Sp*MsrAB may play a role in the structural stability of the protein by hydrogen bond interactions with both the MsrA and MsrB domains. Further biochemical and structural studies will be necessary for verifying the function of the *iloop*. Second, the apparent *K*_m_ value of *Sp*MsrB for the substrate is 20-fold lower than that of *Sp*MsrA, suggesting that *Sp*MsrB forms a complex with its substrate more readily than *Sp*MsrA. Third, in agreement with this kinetic result, we demonstrate the first structure of MsrB complexed with Met residue. As a result of examining the *Sp*MsrB–SHMAEI complex, we propose a model for the catalytic mechanism of MsrB and this model shows how catalytic and recycling cysteine residues are involved in conformational changes to form a disulphide bridge. The structural and subsequent biochemical analysis reveals why *Sp*MsrB can readily form a complex with its substrate and provides insights into the distinct structural nature of the active site of each MsrA and MsrB domain in MsrAB family.

## Experimental procedures

### Cloning, expression and protein purification

The gene encoding the full-length *Sp*MsrAB (residues 1–312) was amplified from the *S. pneumoniae* genome by PCR. The PCR products were then digested with BamHI and XhoI and inserted into pET-28a (+) (Novagen), containing a His-tag. The plasmid was transformed into *E. coli* BL21(DE3). Cells were grown in LB medium and protein expression was induced with 0.5 mM IPTG at 18°C. After induction, the cells were harvested and disrupted by sonication in buffer A [20 mM Tris-HCl (pH 8.0), 100 mM NaCl, 5 mM imidazole and 1 mM DTT]. The lysate was then clarified by centrifugation and was applied to a 5 ml HisTrap column (Amersham Pharmacia). The protein was eluted by linear gradient with buffer A and 5–500 mM imidazole. The His-tag was removed by treatment with thrombin, followed by dialysis overnight at 4°C. The protein was loaded on a 5 ml HiTrap ion exchange column (Amersham Pharmacia) using buffer of 50 mM Tris-HCl (pH 8.0) and 5 mM DTT with a gradient of 0–1.0 M NaCl followed by gel filtration on a HiLoad 26/60 Superdex-200 column (Amersham Pharmacia) using buffer of 25 mM Tris-HCl (pH 8.0), 100 mM NaCl and 5 mM DTT. The purified protein was concentrated to 35 mg ml^−1^. Seleno-Met-labelled *Sp*MsrAB was expressed in *E. coli* B834(DE3) and purified as described above.

### Crystallization and data collection

*Sp*MsrAB crystals suitable for X-ray data collection were grown by the hanging-drop vapour diffusion method at 22°C in [0.1 M MES (pH 5.6), 1% (w/v) PEG 4000 and 0.2 M MgCl_2_]. For cryoprotection, the crystals were soaked in reservoir solution containing 25% ethylene glycol and frozen in cold nitrogen at −173°C. X-ray diffraction was performed at beam line 4A of the Pohang Light Source (PLS), Pohang, Korea. The final X-ray diffraction data were collected with a Quantum 210 CCD detector (Area Detector Systems, Poway, CA). The native *Sp*MsrAB crystal diffracted to 2.4 Å and belongs to the space group P2_1_2_1_2, with unit cell dimenhyqjsions: *a* = 158.5 Å, *b* = 165.5 Å and *c* = 77.3 Å. The SAD data using seleno-Met-labelled *Sp*MsrAB were collected at peak wavelength (0.9795 Å). The data were processed and scaled using HKL2000 (Otwinowski and Minor, 1997).

### Structure determination and refinement

The crystal structure of *Sp*MsrAB was determined by the SAD method. Searching of 12 selenium sites and calculation of phase were carried out with the SOLVE (Terwilliger and Berendzen, 1999; Terwilliger, 2000). The first model was built into 2.7 Å resolution electron density map by using Coot (Emsley and Cowtan, 2004). After refinement with CNS (Brünger *et al*., 1998), the resolution was improved to 2.4 Å. Final refinement, after including a hexa-peptide and solvents, resulted in *R* and *R*_free_ values of 23.9% and 28.2% (for a 10% data sample) respectively. Data collection and refinement statistics are summarized in Table 1. The atomic co-ordinates and structure factors for the *Sp*MsrAB have been deposited in the Protein Data Bank with the accession code 3E0M. A crystal structure of *Bs*MsrB was also determined (Table S1; Park *et al*., 2008) and has been deposited (3E0O). The Protein Data Bank accession codes for other Msr proteins discussed in this article are as follows: *Bt*MsrA (1FVA), *Ec*MsrA (1FF3), *Mt*MsrA (1NWA), *Nm*MsrA (3BQE, 3BQF, 3BQH), *Pt*MsrA (2J89), *Bs*MsrB (1XM0), *Bp*MsrB (3CEZ, 3CXK) and *Ng*MsrB (1L1D).

### Kinetic assays

MsrA and MsrB activities were determined in the presence of DTT using dabsylated Met-SO as substrate. Briefly, a 100 μl reaction mixture contained a buffer of 50 mM sodium phosphate (pH 7.5), 50 mM NaCl and 20 mM DTT, and either 200 μM dabsyl-Met-*S*-SO (for MsrA assays) or dabsyl-Met-*R*-SO (for MsrB assays), and 1 μg of purified protein of *Sp*MsrAB or *Bs*MsrB. To assay for the total Msr activity of *Sp*MsrAB, 400 μM mixed (*R*, *S*) Met-SO was used. The reactions were carried out at 37°C for 30 min and stopped by adding 200 μl of acetonitrile. The dabsyl-Met product was analysed using an HPLC procedure. *K*_m_ and *k*_cat_ values were determined for DTT-dependent reactions from Lineweaver-Burk plots. For determination of *K*_m_, 0.05–0.8 mM dabsyl-Met-*S*-SO was used and 0.05–0.2 mM dabsyl-Met-*R*-SO was used.
