# Supplementary material for: Structural and kinetic analysis of an MsrA–MsrB fusion protein from Streptococcus pneumoniae
Source: Mol Microbiol. 2009 May;72(3):699–709. doi: 10.1111/j.1365-2958.2009.06680.x (PMC2713860; doi:10.1111/j.1365-2958.2009.06680.x)
Supplement: Supplementary file 1 [file mmi0072-0699-SD1.pdf]

Table S1. Data collection and refinement statistics of *B.subtilis* MsrB

	***Bs*MsrB**
	SeMet-peak
Data collection	
Space group	P3
Cell dimension, a, b, c (Å)	136.114 136.114 61.925
Molecules per AU	6
Wavelength (Å)	0.97950
Resolution range (Å)	20.0 – 2.60 (2.69 – 2.60)
No. of measured reflections	1095177
No. of unique reflections	39083 (3846)
Completeness (%)	99.4 (97.9)
Average $I/\sigma(I)$	12.5 (2.8)
R_{merge}[a] (%)	10.1 (43.6)
Refinement	
Resolution range (Å)	20.0 – 2.6
No. of reflections (work/test)	38752 (34867 / 3885)
R_{work}[b] / R_{free}[c]	23.5 / 29.4
B-factors (Å^2) (protein/solvent)	33.2 / 28.9
No. of atoms (protein/ligand/water)	6913 / - / 387
R. m. s. deviations	
Bond lengths (Å)	0.007
Bond angles (deg)	1.3
Ramachandran plot	
Most favored (%)	81.2
Additionally allowed (%)	17.0
Generously allowed (%)	1.7
Disallowed (%)	0.1

[a] $R_{merge} = \sum_h \sum_j |\langle I \rangle_h - I_{hj}| / \sum_h \sum_j |I_{hj}|$, where $\langle I \rangle_h$ is the mean intensity of symmetry-equivalent reflections.
[b] $R_{work} = \sum_h |F_o - F_c| / \sum_h |F_o|$, where F_o and F_c are the observed and calculated structure factor amplitudes of reflection h.
[c] R_{free} is the same as R_{work}, but calculated on the reflections set aside from refinement.

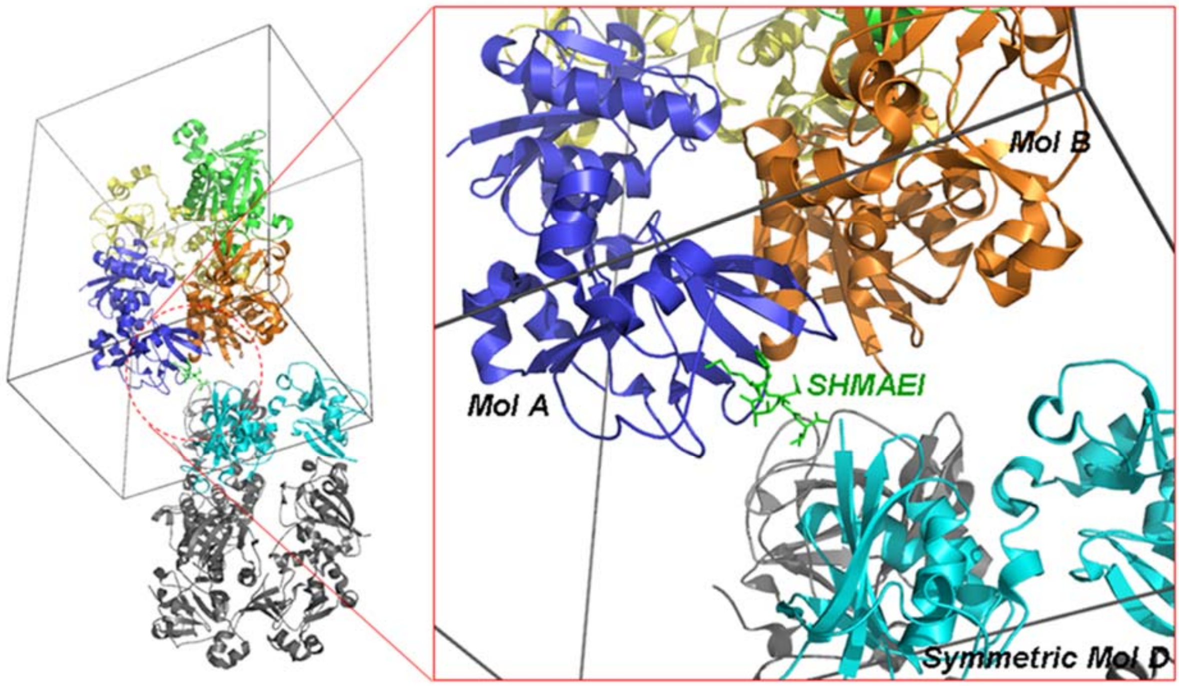

Figure S1. Crystal packing and contact in an asymmetric unit of *Sp*MsrAB

One molecule of *Sp*MsrAB is located on boundary of a crystal lattice. Right panel shows the enlarged figure of left panel. In an asymmetric unit of *Sp*MsrAB, the hexa-peptide (green stick) of a neighboring molecule (Mol D; cyan) that exists in crystallographic symmetry is extended to the active pocket of *Sp*MsrB domain in Mol A (blue).

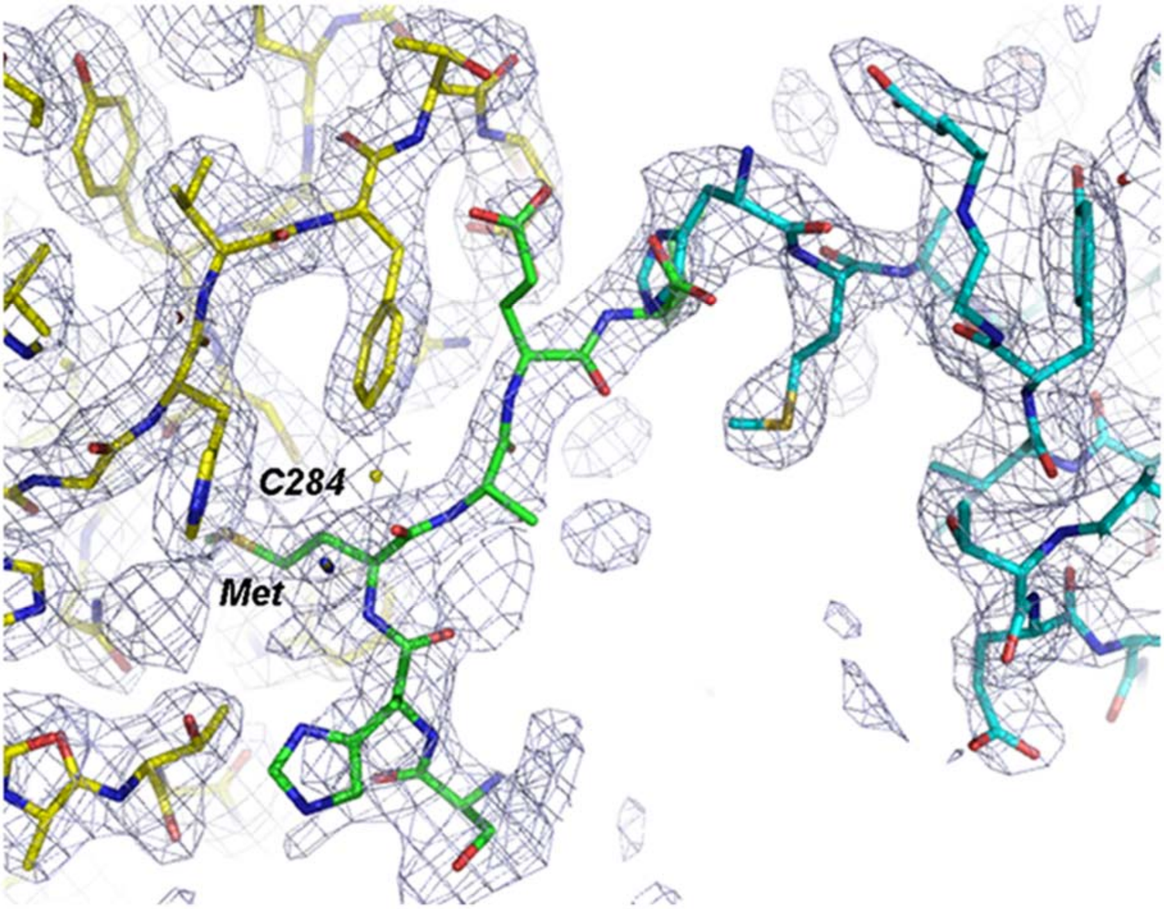

Figure S2. The 2Fo-Fc electron density of active-site of *Sp*MsrB

The 2Fo-Fc electron density of the active-site of MsrB domain including the hexa-peptide from another molecule is shown at 1.0 σ. The Met residue of hexa-peptide binds to the MsrB domain of *Sp*MsrAB.

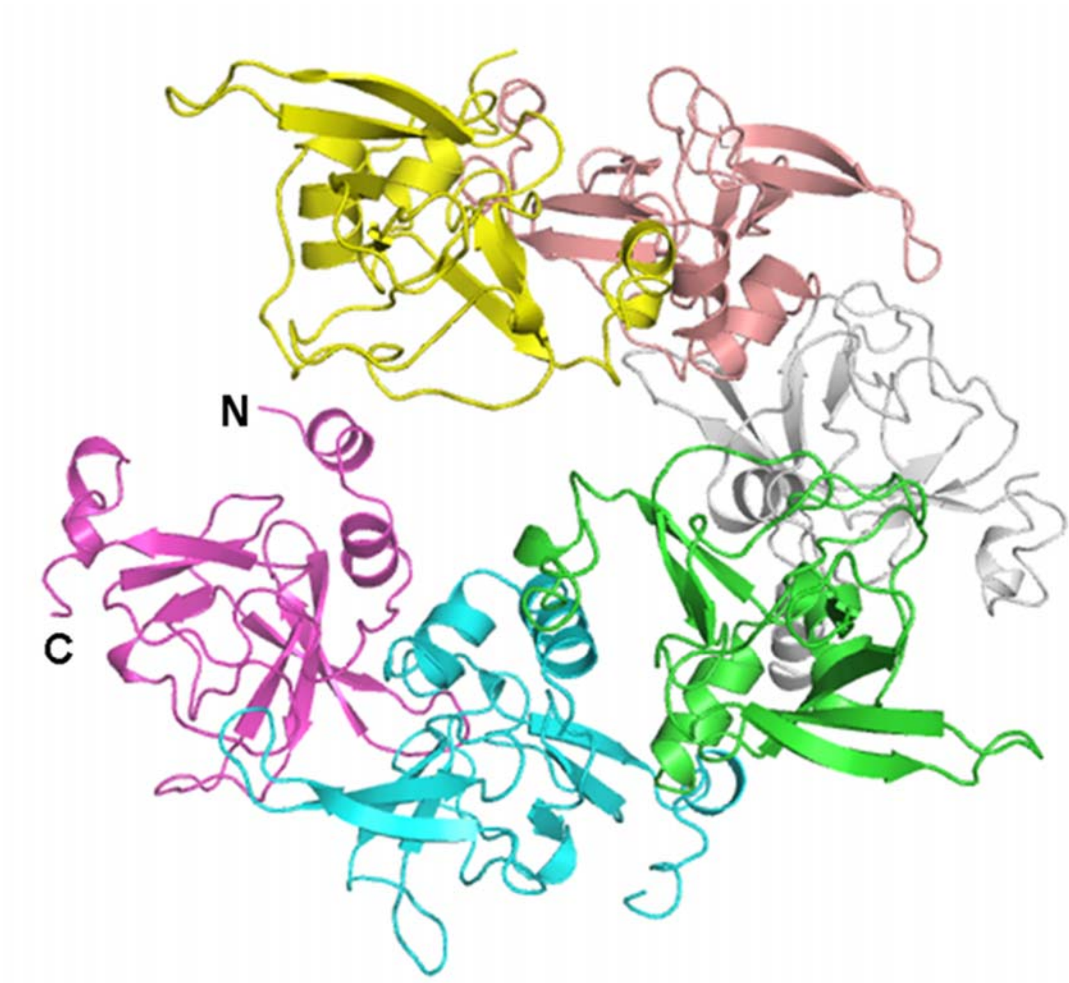

Figure S3. Overall structure of *Bs*MsrB

In the structure of crystallographic hexameric form, all six molecules of *Bs*MsrB show identical conformation in an asymmetric unit. The monomeric crystal structure was found to be identical to the previous NMR structure of 1XM0.

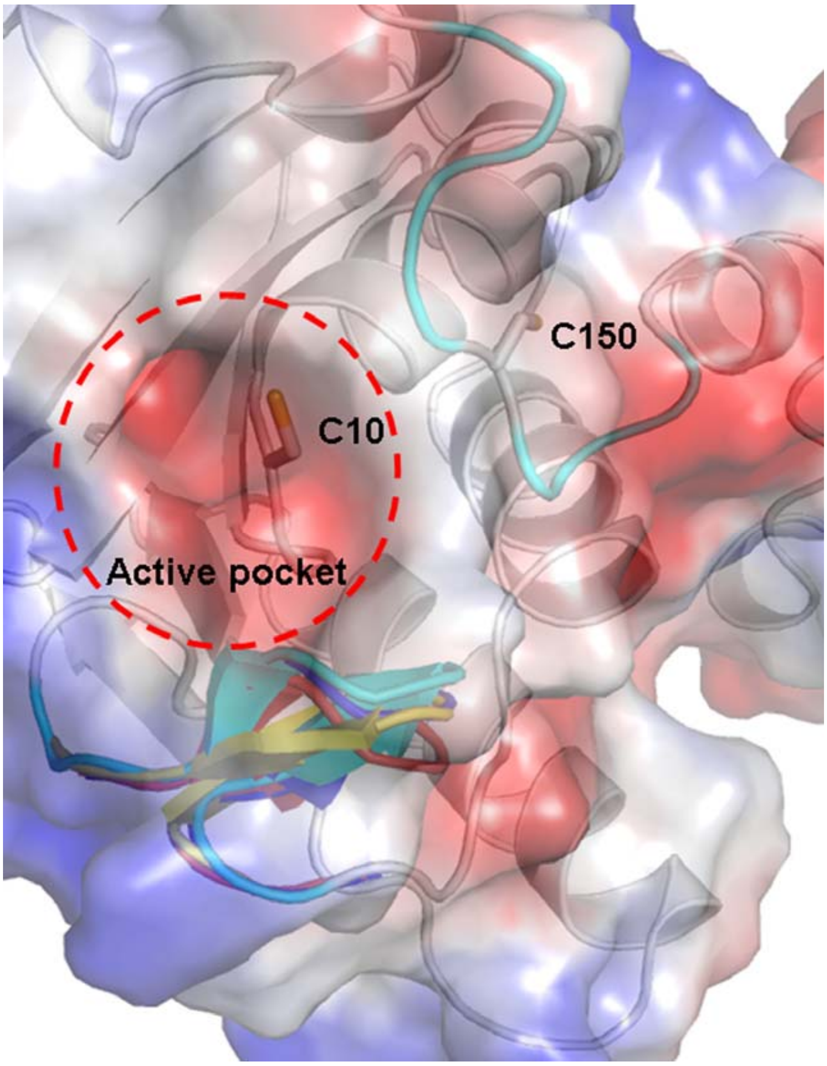

Figure S4. Comparison of the flanked regions of the MsrA active pockets

The flanked loop, including β4 and β5, of the *Sp*MsrA active pocket (cyan) is superposed with those of the reduced *Nm*MsrA (red; 3BQE) and the complexed form with a Met-SO substrate (blue; 3BQF) and the complexed *Mt*MsrA with a Met (yellow; 1NWA). The RMS deviations for Cα of the flanked loops are as follows: *Sp*MsrA-*Nm*MsrA reduced, 3.4 Å; *Sp*MsrA-*Nm*MsrA complexed, 3.2 Å; *Sp*MsrA-*Mt*MsrA complexed, 0.7 Å.

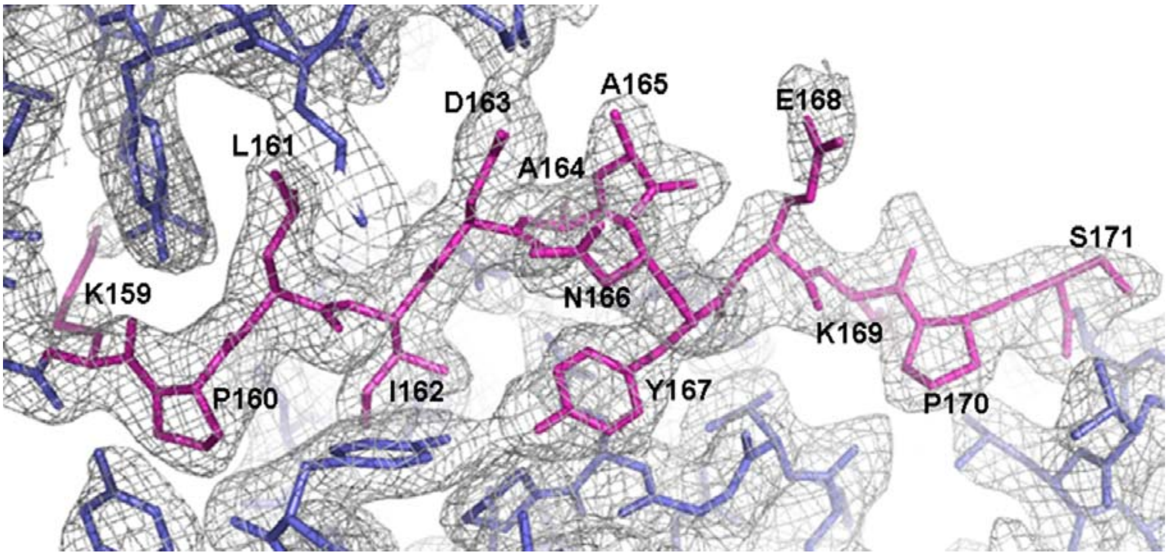

Figure S5. The 2Fo-Fc electron density of the linker region at 1.0 σ

The linker region of Mol A (conformation 1) is presented with its density map. The color scheme is same as in Fig. 7A (Con 1).
